# Targeting autophagy enhances atezolizumab-induced mitochondria-related apoptosis in osteosarcoma

**DOI:** 10.1038/s41419-021-03449-6

**Published:** 2021-02-08

**Authors:** Zhuochao Liu, Hongyi Wang, Chuanzhen Hu, Chuanlong Wu, Jun Wang, Fangqiong Hu, Yucheng Fu, Junxiang Wen, Weibin Zhang

**Affiliations:** 1 Department of Orthopedics, Shanghai Key Laboratory for Prevention and Treatment of Bone and Joint Diseases, Shanghai Institute of Traumatology and Orthopedics, Ruijin Hospital, Shanghai Jiao Tong University School of Medicine, Shanghai, China; 2 Department of Orthopaedics, Shanghai Tenth People’s Hospital, Tongji University, Shanghai, 200072 China

**Keywords:** Targeted therapies, Sarcoma

## Abstract

In this study, we identified the multifaceted effects of atezolizumab, a specific monoclonal antibody against PD-L1, in tumor suppression except for restoring antitumor immunity, and investigated the promising ways to improve its efficacy. Atezolizumab could inhibit the proliferation and induce immune-independent apoptosis of osteosarcoma cells. With further exploration, we found that atezolizumab could impair mitochondria of osteosarcoma cells, resulting in increased release of reactive oxygen species and cytochrome-c, eventually leading to mitochondrial-related apoptosis via activating JNK pathway. Nevertheless, the excessive release of reactive oxygen species also activated the protective autophagy of osteosarcoma cells. Therefore, when we combined atezolizumab with autophagy inhibitors, the cytotoxic effect of atezolizumab on osteosarcoma cells was significantly enhanced in vitro. Further in vivo experiments also confirmed that atezolizumab combined with chloroquine achieved the most significant antitumor effect. Taken together, our study indicates that atezolizumab can induce mitochondrial-related apoptosis and protective autophagy independently of the immune system, and targeting autophagy is a promising combinatorial approach to amplify its cytotoxicity.

## Introduction

Osteosarcoma (OS) is the most common primary malignant tumor of bone in adolescents, and the incidence is 4.675 per million in people under 19 years of age^1^. Despite its rarity, OS is the third most common cancer in adolescence, with strong local invasive and metastatic abilities, placing a heavy burden on society and family^2,3^. Although surgical resection techniques have been greatly improved in recent decades, medicine treatment still relies on the same drugs as in the early 1970s, and the 5-year survival rate has not improved significantly since then^4^. Therefore, it is urgent to find new and effective medicines to further improve the survival rate of OS patients.

Programmed cell death ligand-1 (PD-L1), known as an immune checkpoint in the B7-H family, promotes tumor progression by suppressing the antitumor immunity of T cells via binding to programmed death-1 (PD-1)^5,6^. Antibodies against PD-L1 have achieved remarkable success in the treatment of advanced tumors such as melanoma, non-small-cell lung cancer, urothelial carcinoma, and breast cancer^7–10^. Our previous study suggested that immunotherapy targeted PD-L1 could be an effective approach in treating OS^11,12^. Besides, most of the localized OS present tumor-infiltrating lymphocytes (CD3^+^ lymphocytes: 90%; CD8^+^ lymphocytes: 86%) and the positive rate of PD-L1 in OS is relatively high^13,14^. But a recent clinical trial (SARC 028 study) shows that the response rate of pembrolizumab in the treatment of advanced OS is poor (5%)^15^, which triggers us to gain a wide insight into its antitumor approach and decipher the reason for the limited response rate.

The principal mechanism of antibodies against PD-L1 is thought to block tumor-extrinsic PD-L1 binding to PD-1^+^ lymphocytes to restore the antitumor immunity^16^. Considering this mechanism, most of the research focuses on the extrinsic effect of blocking PD-1/PD-L1 axis, particularly on T cells. However, recent works show that PD-L1 expression in tumor cells also has direct effects on preventing it from apoptosis, sustaining its stemness, regulating its metabolism, and promoting autophagy^17–20^. Hence, PD-L1 directly plays a pro-survival role in tumor cells, but few studies have investigated the direct effects of blocking PD-L1 on tumor cells themselves.

Atezolizumab, a specific monoclonal antibody against PD-L1, can inhibit the combination between PD-L1 and PD-1^21^. In this study, we observed the immune-independent antitumor effect of atezolizumab. Atezolizumab could directly damage mitochondria in OS cells, increasing the release of reactive oxygen species (ROS) and cytochrome-c (cyto-c), and ultimately lead to mitochondrial-related apoptosis. However, the excessive release of ROS could also induce autophagy. And the combination of autophagy inhibitors with atezolizumab could enhance the cytotoxicity of atezolizumab on OS cells both in vitro and in vivo. Therefore, we propose that targeting atezolizumab-induced autophagy may be a potential therapeutic approach to enhance the efficacy of atezolizumab in OS.

## Materials and methods

### Cell culture and reagents

Human OS cell lines HOS and 143B were obtained from ATCC (Manassas, VA, USA). All cells were cultured in high glucose Dulbecco’s modified Eagle’s medium supplemented with 10% fetal bovine serum and 1% penicillin-streptomycin at 37 °C. Different concentrations of atezolizumab (A2004, Selleck, USA) were added to the medium. SP600125, a JNK inhibitor (S1460, Selleck, USA) was added to the medium at 10 μM. Mito-TEMPO (SML0737, Sigma, USA) was added to the medium at 10 μM as a mitochondria-targeted antioxidant. To inhibit the autophagy induced by atezolizumab, OS cells were pre-treated with chloroquine (CQ) (C6628, Sigma, USA) for 16 h at 10 μM, and then atezolizumab and CQ were added together for another 24 h. We chose the OS cells treated with an apoptotic inducer (C0005, Beyotime, China) at 1:1000 for 24 h as the positive control of apoptosis-related proteins staining. And the positive control for autophagy was by starving the cells for 12 h.

### Cell viability assays and colony formation assay

The cytotoxicity of atezolizuamb on HOS and 143B was estimated using Cell Counting Kit-8 (CCK-8) (Dojindo, Japan). As we previously described^12^, OS cells were seeded at a density of 5 × 10^3^ cells/well in 96-well plates before examination. Then, OS cells were treated with atezolizumab at 0, 2.5, 5, 10, 20, and 40 μg/ml for 24 h. For the colony formation assay, 1 × 10^3^ OS cells were plated in six-well plates and cultured with different concentrations of atezolizumab at 37 °C for 2 weeks. After staining with 0.1% crystal violet for 15 min, the number of colonies was visualized and quantified.

### Quantitative RT-PCR analysis

mRNA expression of Atg-5 was analyzed by qRT-PCR. The primer sequence of Atg-5 forward: 5′-GATGGGATTGCAAAATGACAGA-3′ and reverse: 5′-GAAAGGTCTTTCAGTCGTTGTC-3′. qRT-PCR was performed using an ABI 7500 detection system according to the manufacturers’ instructions. The expression of target genes was calculated using 2^−△△CT^.

### Transmission electron microscopy

After the treatment of atezolizumab for 24 h, OS cells were washed with PBS. Subsequently fixed with 2.5% glutaraldehyde solution overnight at 4 °C. After further fixation in 1% osmium tetroxide for 2 h and gradual dehydration in alcohols, the samples were embedded in Epon resin. Ultrathin sections (50 nm) were observed on a Hitachi electron microscope equipped with a digital camera.

### Mitochondrial membrane potential detection

The mitochondrial membrane potential was detected with mitochondrial membrane potential assay kit with JC-1 (C2006, Beyotime, China). Treated OS cells with atezolizumab for 24 h, and replaced the medium with fresh medium. Then incubated with JC-1 working solution in a CO_2_ incubator for 20 min. After incubation, removed the staining solution and washed with JC-1 staining buffer twice. OS cells were imaged using confocal microscopy and the change in mitochondrial membrane potential (Δψm) was reflected by the ratio of green fluorescence to red fluorescence (ratio of JC-1 monomers/JC-1 aggregates).

### Autophagy flux analysis

HOS and 143B were transfected with recombinant viral vectors encoding GFP-RFP- LC3 (Genomeditech, China). 72 h after transfection, HOS and 143B were treated with puromycin at 5 μg/ml to construct OS cells continuously reporting autophagy activity. Then the transfected OS cells were treated with atezolizumab with or without mito-TEMPO, and then the activation of autophagy was detected via confocal microscopy.

### Confocal microscopy

OS cells were fixed using 4% paraformaldehyde (PFA) for 15 min and then permeabilized with 0.1% Triton X-100 for 10 min at room temperature. After blocked with blocking buffer (P0260, Beyotime, China) for 10 min at room temperature, the slices were incubated with antibodies against cleaved-caspase-3 (ab13847, Abcam, USA), cleaved-caspase-9 (ab202068, Abcam, USA) or LC3B (ab48394, Abcam, USA) overnight at 4 °C. The slices were imaged using confocal microscopy.

### Western blot

Western blot was performed as previously described^22^. The blots were incubated with antibodies against cleaved-caspase-3 (ab13847, Abcam, USA), cleaved-caspase-9 (ab202068, Abcam, USA), Bax (ab32503, Abcam, USA), Bcl-2 (ab59348, Abcam, USA), cytochrome-c (ab133504, Abcam, USA), phospho-JNK (5599, CST, USA), JNK (9252, CST, USA), LC3B (ab48394, Abcam, USA), Beclin-1 (ab207612, Abcam, USA), P62 (ab109012, Abcam, USA) or Atg-5 (ab108327, Abcam, USA) at 4 °C overnight. Protein amounts were determined by densitometric analysis and normalized to β-actin (3700, CST, USA).

### Xenograft tumor model, immunochemistry, and TUNEL assay

Six-week-old male severe combined immunodeficiency mice were used for tumor implantation. HOS (2 × 10^6^ cells/mouse) was subcutaneously implanted into the dorsal gluteal region. The mice were randomly divided into four groups: control group (PBS only), CQ treatment group (CQ only), atezolizumab treatment group (atezolizumab only), and atezolizumab combined with CQ group (*N* = 5 per group). Atezolizumab (10 mg/kg) and CQ (50 mg/kg) were administered everyday intraperitoneally by a technician who is blind to the identity of each group. Tumor size was measured every 3 days and calculated using the equation (length × width^2^)/2, and administrations began when the tumor volume reached 100 mm^3^. Mice were sacrificed 3 weeks after OS cells inoculation, and xenograft tumors were digested using type IV collagenase (1 mg/ml) and DNase I (0.2 mg/ml) (Sigma-Aldrich) into a single-cell suspension for ROS detection or fixed with 4% PFA for immunohistochemistry (IHC) and TUNEL staining. The IHC staining of Ki-67 was performed using the Super Sensitive IHC Detection System Kit (BD5001, Bioworld) according to the manufactures’ instructions. Slides were incubated with anti-Ki-67 (ab16667, Abcam, USA) overnight at 4 °C. The positive rate of Ki-67 was counted by three pathologists who were blind to the characteristics of each slide. The TUNEL assay was carried out through a TUNEL Apoptosis Assay Kit (C1090, Beyotime, China) according to manufacturers’ instructions. This protocol was approved by the Ethics Committee of Ruijin Hospital, affiliated with Shanghai Jiaotong University School of Medicine.

### Flow cytometry assay

OS cells were digested by 0.25% trypsin with EDTA after treatments and incubated with an Annexin V-FITC Apoptosis Detection Kit (V13241, Invitrogen, USA) according to manufacturers’ instructions. After incubation, OS cells were subjected to flow cytometry and analyzed with CytExpert software (Beckman, USA).

### RNA interference

Small interfering RNA (siRNA) targeting Atg-5 were purchased from GenePharma (Shanghai, China). The siRNA Sequence of Atg-5 is (5′-GGAGTCACAGCTCTTCCTT-3′). HOS was transfected with Atg-5 siRNA using Lipofectamine 3000 (Thermo Fisher, USA) as described previously^23^.

### Oxidative stress determination

The state of redox in xenograft tumors and OS cells were determined by measuring the amount of ROS with ROS assay kit (S0033, Beyotime, China), the amount of malondialdehyde (MDA) with Lipid peroxidation MDA assay kit (S0121, Beyotime, China), the amount of total antioxidant capacity (T-AOC) with T-AOC assay kit (S0121, Beyotime, China), and the amount of superoxide dismutase (SOD) with Total SOD assay kit (S0109, Beyotime, China) according to manufacturers’ instructions. The level of ROS was detected by CytoFlex S (Beckman, USA) and the others were detected by Infinite pro 200 (Tecan, Switzerland). The levels of MDA and T-AOC were expressed as μmol/g protein. And the level of SOD was expressed as U/g protein.

### Statistical analysis

All data are from at least three independent experiments and are expressed as mean ± standard deviation. And all data are in a normal distribution. Statistical differences between groups were estimated using a Student’s *t* test or one-way ANOVA. Statistical analyses were performed using GraphPad Prism 5.0.

## Result

### Atezolizumab inhibits proliferation and induces immune-independent apoptosis of osteosarcoma cells

**Fig. 1:**
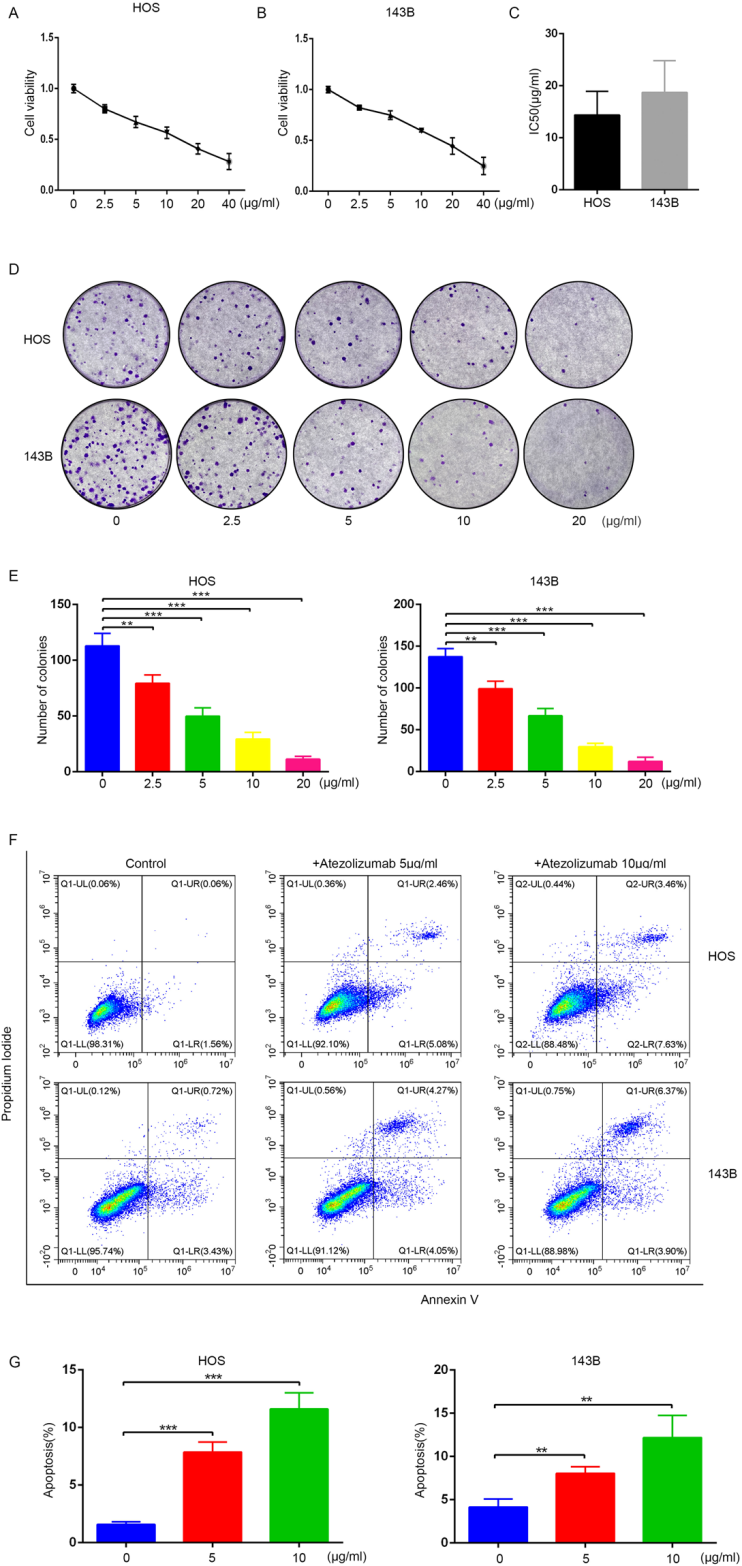
Atezolizumab inhibits proliferation and promotes immune-independent apoptosis of OS cells in vitro. **A**, **B** Proliferation of HOS and 143B was detected by CCK-8 assay after the treatment of atezolizumab with different concentrations; **C** IC_50_ of HOS and 143B to atezolizumab; **D**, **E** Proliferation of HOS and 143B was detected by colony formation assay after the treatment of atezolizumab; **F**, **G** Apoptosis of HOS and 143B was detected by flow cytometry after the treatment of atezolizumab. All data are from at least three independent experiments and are presented as the means ± SD. *P < 0.05; **P < 0.01; ***P < 0.001.

In order to identify the effect of atezolizumab exerted on the proliferation of OS cells, different concentrations (0, 2.5, 5, 10, 20, and 40 µg/ml) of atezolizumab were applied to human OS cell lines HOS and 143B for 24 h. As shown in Fig. 1A, B, the proliferation of HOS and 143B both were inhibited by atezolizumab in a dose-dependent manner. The IC50 values of HOS and 143B were also shown in Fig. 1C. Besides, colony formation assay was performed to confirm the role of atezolizumab in inhibiting OS cell proliferation, and the number of colonies was significantly reduced after the treatment of atezolizumab (Fig. 1D, E).

Inducing apoptosis is an important mode for many drugs to exert cytotoxicity. To further identify whether atezolizumab has a proapoptotic effect on OS cells in vitro, we performed flow cytometry to detect tumor cell apoptosis. After the incubation of HOS and 143B with atezolizumab for 24 h, the percentage of apoptotic cells increased dramatically compared to the control group (Fig. 1F, G, HOS 1.58% vs. 11.61%, *P* = 0.0003; 143B 4.15% vs. 12.2%, *P* = 0.0068). Moreover, the proapoptotic effect of atezolizumab was also dose-dependent as the percentage of apoptotic cells increased gradually with increasing concentration (Fig. 1F, G). Taken together, these results indicate that atezolizumab can inhibit the proliferation and induce apoptosis of OS cells in a dose-dependent manner in vitro.

### Atezolizumab impairs the function of mitochondria to cause the imbalance between oxidants and antioxidants

**Fig. 2:**
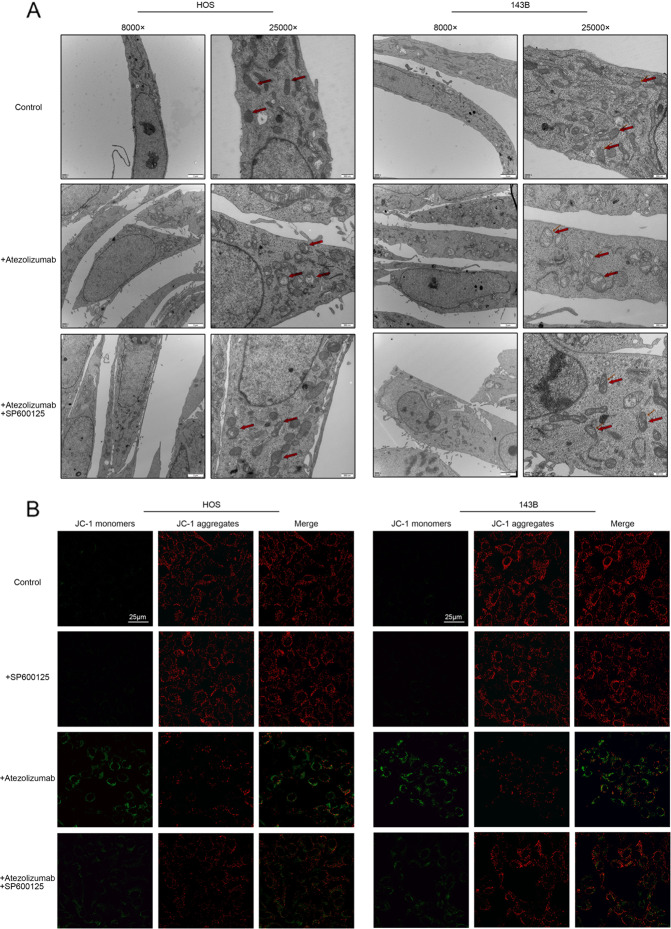

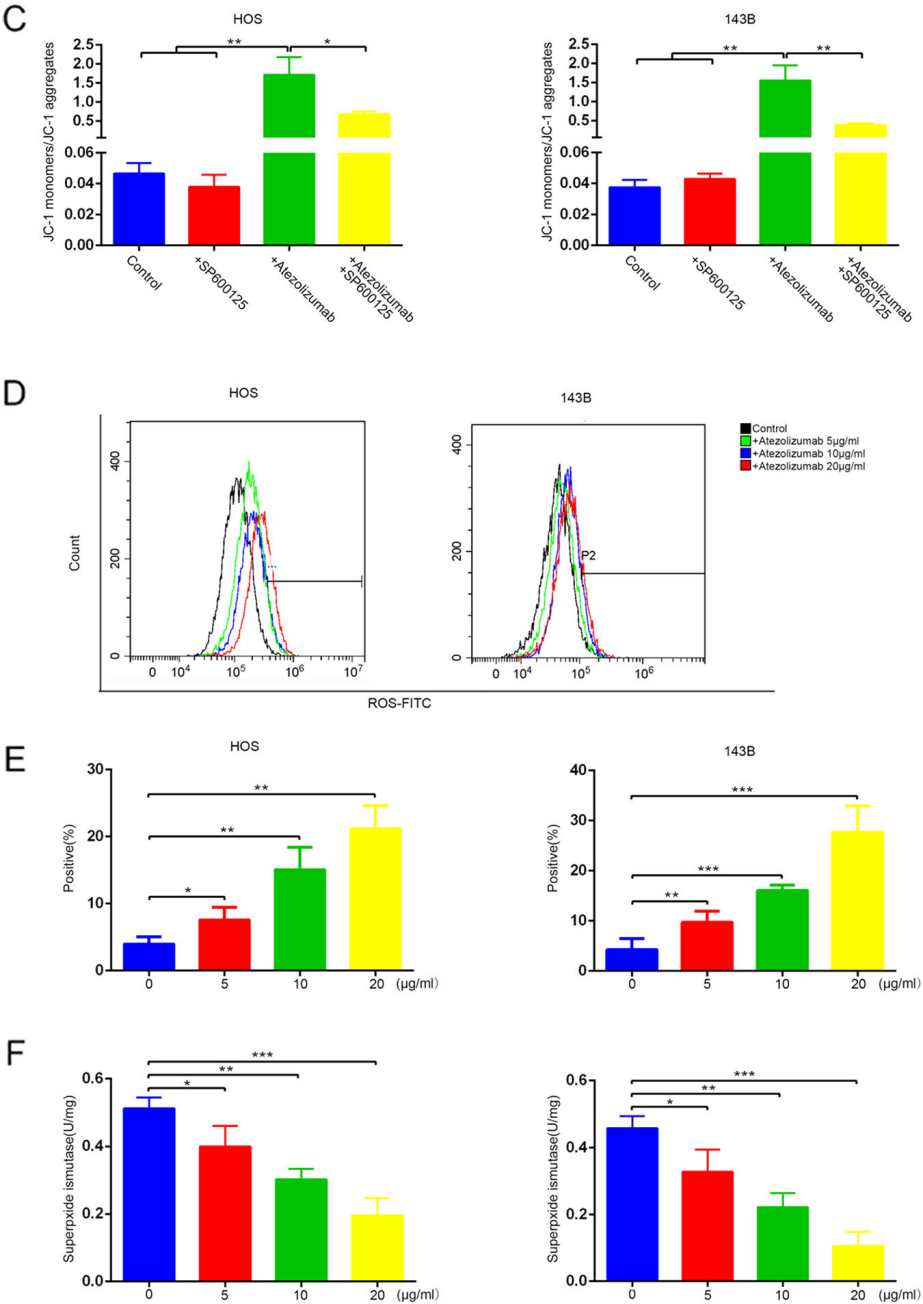
Atezolizumab impairs the function of mitochondria to cause the imbalance between oxidants and antioxidants. **A** Transmission electron microscopy of HOS and 143B after the treatment of atezolizumab (10 µg/ml) (red arrows point to mitochondria); **B** Mitochondrial membrane potential of HOS and 143B was detected by JC-1 staining after the treatment of atezolizumab (normal OS cells which have high ΔΨm show strong red fluorescence and weak green fluorescence); **C** Quantification of JC-1 staining in HOS and 143B; **D**, **E** Release of ROS in HOS and 143B was detected by flow cytometry after the treatment of atezolizumab; **F** Amount of SOD in HOS and 143B was detected by the total SOD assay kit. All data are from at least three independent experiments and are presented as the means ± SD. *P < 0.05; **P < 0.01; ***P < 0.001.

Based on the above results, we could draw a preliminary conclusion that atezolizumab could inhibit the proliferation of OS cells directly independent of the immune system. But the specific mechanism remains uncertain. To explore the underlying mechanisms, we tested the change in mitochondria, the main source of energy for cells. Morphologically, the mitochondria were vacuolated and mitochondria crest swelled after the addition of atezolizumab (Fig. 2A). Functionally, we detected the loss of the mitochondrial membrane potential (ΔΨm) as the fluorescence ratio of JC-1 monomers to JC-1 aggregates increased after the stimulation of atezolizumab (Fig. 2B, C). The damage to mitochondria also caused the excessive release of ROS. As the result shown in Fig. 2D, E, the positive rate of ROS in OS cells increased as the concentration of atezolizumab increased. Meantime, the amount of SOD, an antioxidant enzyme, reduced significantly after atezolizumab stimulation (Fig. 2F). These findings strongly indicate that atezolizumab can cause mitochondrial damage to induce the imbalance between oxidants and antioxidants.

### Atezolizumab induces mitochondria-related apoptosis of osteosarcoma cells by activating JNK pathway

**Fig. 3:**
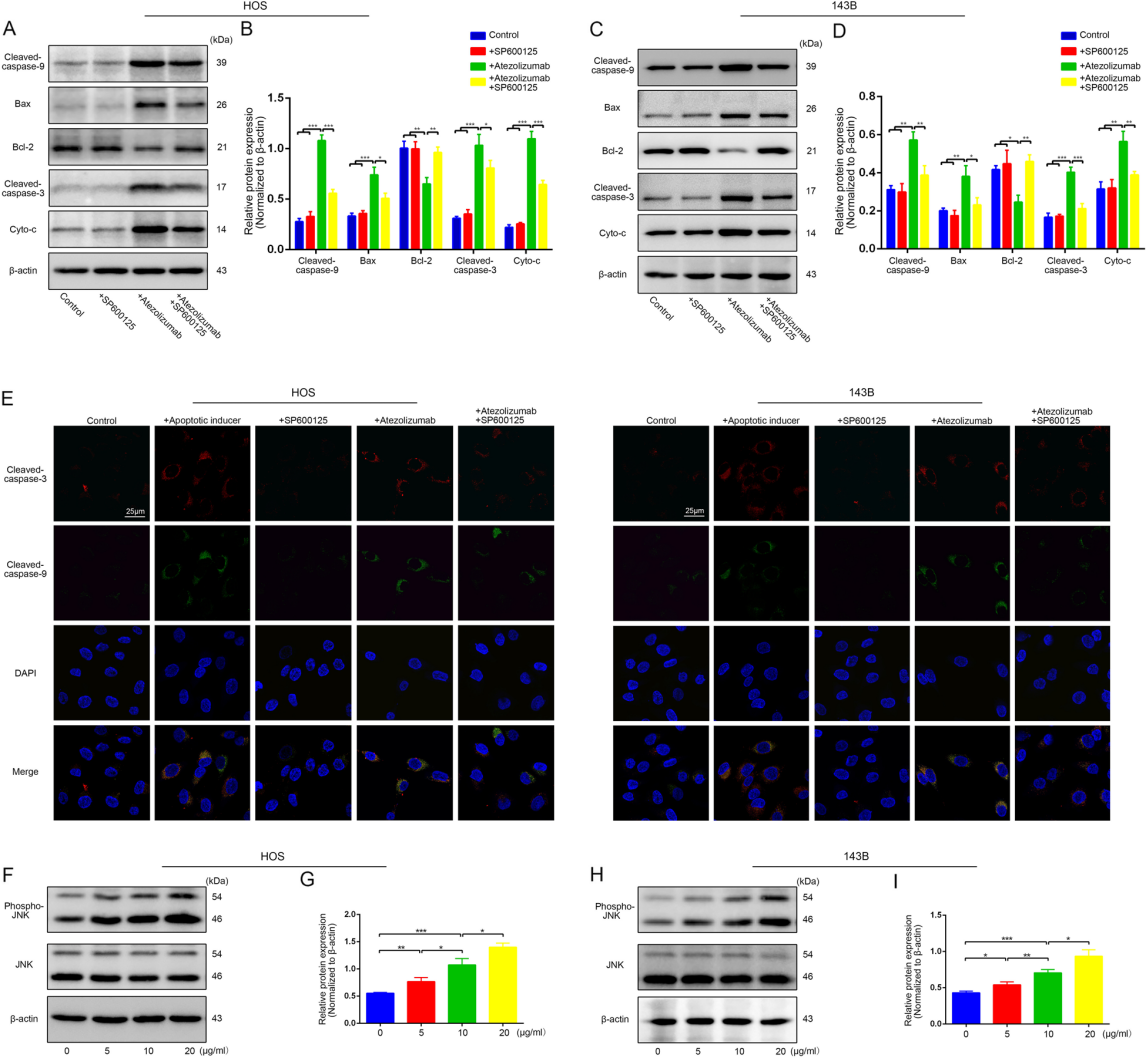
Atezolizumab induces the mitochondria-related apoptosis of OS cells by activating JNK pathway. **A**–**D** Expression of mitochondria-related apoptosis protein in HOS and 143B was evaluated by western blot after the treatment of atezolizumab (10 µg/ml) with or without SP600125 (10 μM); **E** Representative immunofluorescence staining images of cleaved-caspase-9 (green) and cleaved-caspase-3 (red) in HOS and 143B after the treatment of atezolizumab with or without SP600125; **F**–**I** Expression of phospho-JNK and JNK in HOS and 143B was detected by western blot after the treatment of atezolizumab with different concentrations. All data are from at least three independent experiments and are presented as the means ± SD. *P < 0.05; **P < 0.01; ***P < 0.001.

Mitochondria are not only the main energy supply organelles in cells, but also play an indispensable role in the regulation of apoptosis. As the results shown in Fig. 3A–D, with the damage of mitochondria, there was also an increased release of cyto-c. Along with the process, there was the activation of caspase-9 followed by caspase-3 activation, leading to mitochondria-related apoptosis (Fig. 3A–E). The activation of JNK pathway was closely related to the mitochondria-related apoptosis, and with the apoptosis of OS cells increased, there was also a simultaneous activation of JNK pathway (Fig. 3F–I). But with the addition of SP600125, a specific JNK pathway inhibitor, the morphological as well as the functional damages of mitochondria induced by atezolizumab were alleviated (Fig. 2A–C). At the same time, with the blockade of JNK pathway, the proapoptotic effect of atezolizumab to OS cells was also weakened, as the expression of cleaved caspase-3 and cleaved caspase-9 decreased (Fig. 3A–E). These findings demonstrate that atezolizumab can induce mitochondria-related apoptosis in OS cells by activating JNK pathway.

### Atezolizumab induces autophagy in osteosarcoma cells

**Fig. 4:**
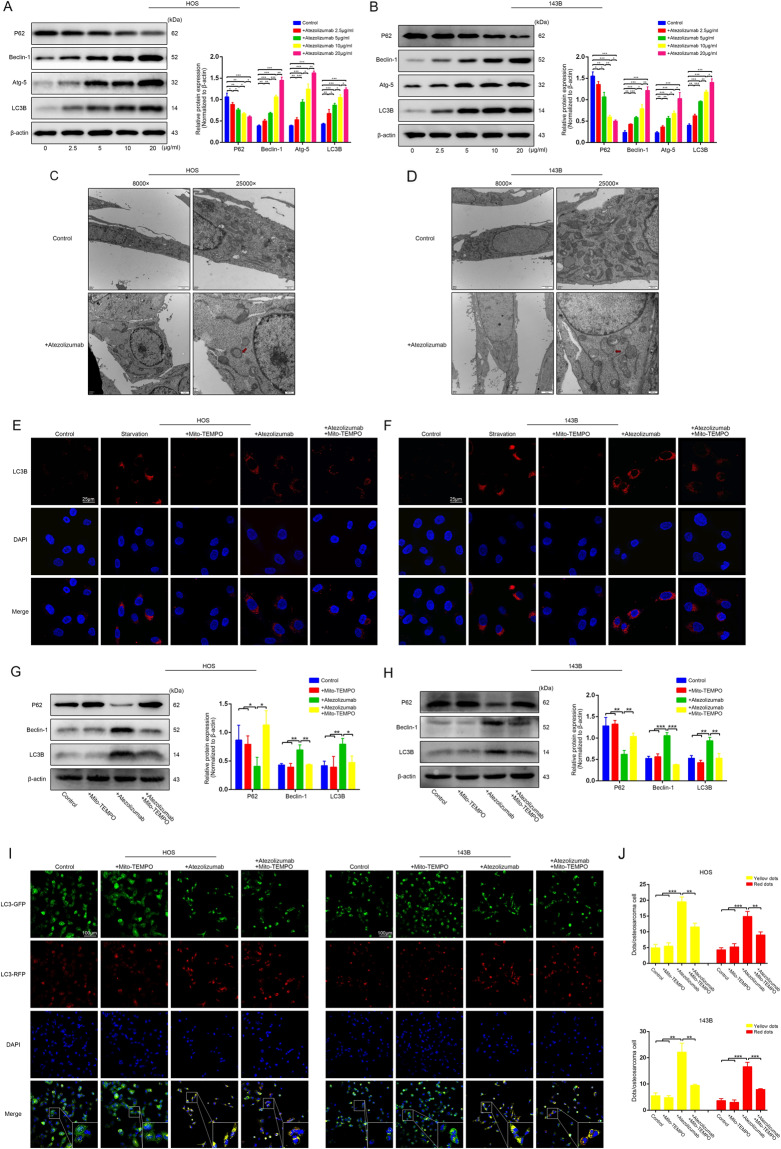
Atezolizumab induces autophagy in OS cells. **A**, **B** Expression of autophagy-related protein in HOS and 143B was evaluated by western blot after the treatment of atezolizumab with different concentrations; **C**, **D** Transmission electron microscopy of HOS and 143B after the treatment of atezolizumab (10 µg/ml) for 24 h; **E**, **F** Representative immunofluorescence staining images of LC3B in HOS and 143B after the treatment of atezolizumab (10 µg/ml) with or without mito-TEMPO (10 μM); **G**, **H** Expression of autophagy-related protein in HOS and 143B was evaluated by western blot after the treatment of atezolizumab with or without mito-TEMPO; **I**, **J** Representative images and analysis of GFP-RFP-LC3 transfected HOS and 143B after the treatment of atezolizumab with or without mito-TEMPO. All data are from at least three independent experiments and are presented as the means ± SD. *P < 0.05; **P < 0.01; ***P < 0.001.

The impairment of mitochondrial can lead to increased ROS release, and excessive release of ROS can also activate autophagy, which is essential for maintaining intracellular homeostasis. To clarify whether atezolizumab could induce autophagy in OS cells, we detected the expression of autophagy substrate (P62) in HOS and 143B and found that the expression of P62 in these two cell lines both decreased gradually with the increment of atezolizumab concentration, while the other autophagy-related proteins (such as Beclin-1, Atg-5, and LC3B) showed a crosscurrent (Fig. 4A, B). Additionally, according to the results of transmission electron microscopy, the number of autophagosomes in OS cells increased after the treatment of atezolizumab (Fig. 4C, D). Furthermore, we observed that the expression of LC3B in situ in OS cells in the atezolizumab treated group was significantly higher than that in the control group (Fig. 4E, F). The activation of autophagy was closely related to the mitochondrial-derived ROS because when we added mito-TEMPO, a mitochondrial-targeted antioxidant, the activation of autophagy induced by atezolizumab was partially relieved (Fig. 4E–H). Autophagic flux was also detected in HOS and 143B after atezolizumab treatment. After viral transfection, GFP-RFP-LC3 fusion protein was dispersed in the cytoplasm equally, and the autophagosomes were yellow spots (overlay of green fluorescence and red fluorescence). In autolysosomes, RFP was stably expressed while GFP quenching, so autolysosomes only displayed red spots. As shown in Fig. 4I, J, after the treatment of atezolizumab for 24 h, the numbers of both yellow and red spots increased dramatically in the atezolizumab group compared to the control group both in HOS and 143B, which mean atezolizumab activated autophagy in OS cells. And the decrease of yellow and red spots after the addition of mito-TEMPO further confirmed that mitochondrial-derived ROS played an important role in atezolizumab-induced autophagy (Fig. 4I, J). Thus, we propose that atezolizumab can cause excessive release of mitochondrial-derived ROS and thereby activate autophagy in OS cells.

### Inhibition of autophagy enhances atezolizumab-induced apoptosis in osteosarcoma cells

**Fig. 5:**
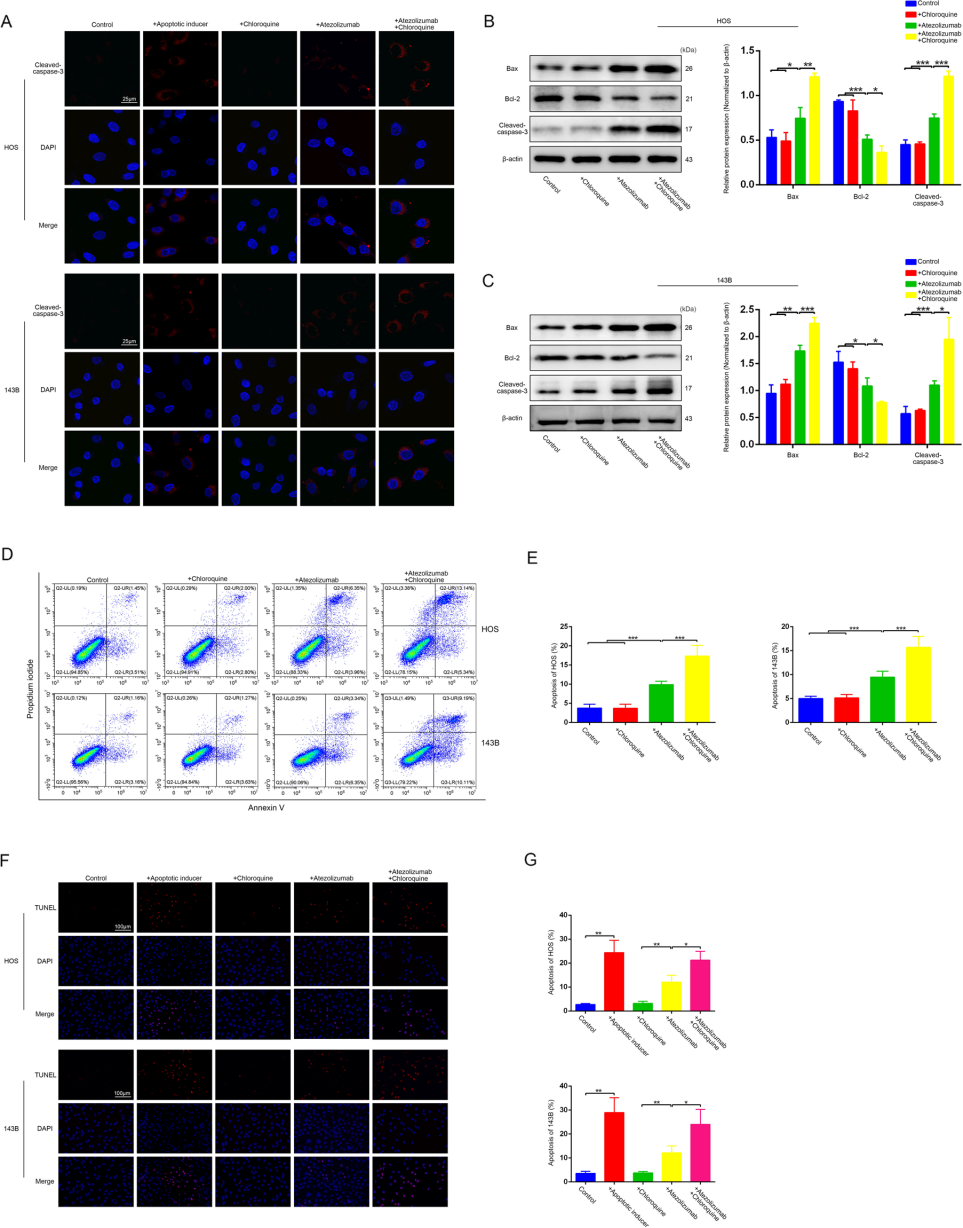
Inhibition of autophagy by CQ enhances atezolizumab-induced apoptosis in OS cells. **A** Representative immunofluorescence staining images of cleaved-caspase-3 in HOS and 143B after the treatment of atezolizumab (10 µg/ml) with or without CQ (10 μM); **B**, **C** Expression of apoptosis-related protein in HOS and 143B was detected by western blot after the treatment of atezolizumab with or without CQ; **D**, **E** Apoptosis of HOS and 143B was detected by flow cytometry after the treatment of atezolizumab with or without CQ; **F**, **G** Representative images and quantification of TUNEL staining in HOS and 143B after the treatment of atezolizumab with or without CQ. All data are from at least three independent experiments and are presented as the means ± SD. *P < 0.05; **P < 0.01; ***P < 0.001.

Autophagy plays a variety of roles in tumorigenesis, as it can both promote and inhibit tumor growth. To explore the exact effect of atezolizumab-induced autophagy on cytotoxicity of atezolizumab, we added CQ, an autophagy inhibitor, along with atezolizumab treatment. Compared with the group in which only atezolizumab was added, atezolizumab combined with CQ could potentiate the activation of caspase-3 (Fig. 5A). The other apoptosis-related proteins also showed similar trends, there was an increase in proapoptotic protein (Bax and cleaved-caspase-3) and a decrease in anti-apoptotic protein (Bcl-2) (Fig. 5B, C). To further confirm whether CQ could enhance the proapoptotic effect of atezolizumab, we performed flow cytometry and TUNEL staining. As shown in Fig. 5D–G, the combination of atezolizumab and CQ did enhance the apoptosis of OS cells induced by atezolizumab.

**Fig. 6:**
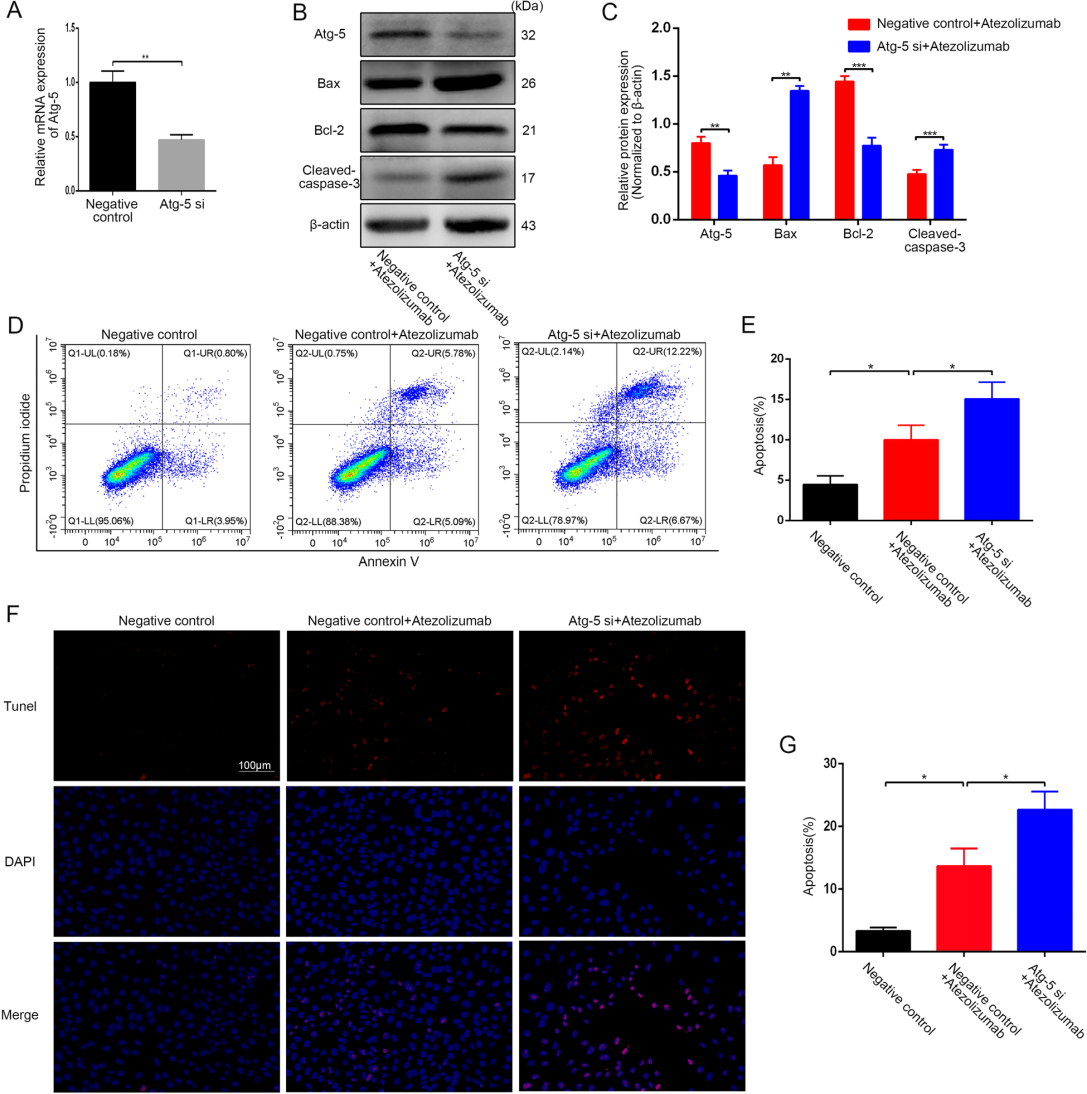
Inhibition of autophagy by Atg-5 siRNA enhances atezolizumab-induced apoptosis in OS cells. **A** Expression of Atg-5 in HOS was detected by qRT-PCR after siRNA transfection; **B**, **C** Expression of Atg-5, Bax, Bcl-2, and cleaved-caspase-3 in HOS was detected by western blot after siRNA transfection; **D**, **E** Apoptosis of HOS was detected by flow cytometry after Atg-5 siRNA transfection; **F**, **G** Representative images and quantification of TUNEL staining in HOS after Atg-5 siRNA transfection. All data are from at least three independent experiments and are presented as the means ± SD. *P < 0.05; **P < 0.01; ***P < 0.001.

Subsequently, we knocked down the autophagy-related gene 5 (Atg-5) in HOS by siRNA to block autophagy in OS cells in another way (Fig. 6A). And we found that the knockdown of Atg-5 could enhance the expression of Bax and cleaved-caspase-3 while reducing the expression of Bcl-2 (Fig. 6B, C). Furthermore, blocking autophagy via Atg-5 siRNA also promoted atezolizumab-induced OS cell apoptosis (Fig. 6D, E), which was subsequently confirmed by TUNEL staining (Fig. 6F, G). These data suggest that atezolizumab can induce protective autophagy in OS cells, and atezolizumab combined with autophagy inhibitors can enhance the cytotoxicity of atezolizumab on OS cells in vitro.

### Tumor suppression effect of atezolizumab is enhanced by chloroquine in vivo

**Fig. 7:**
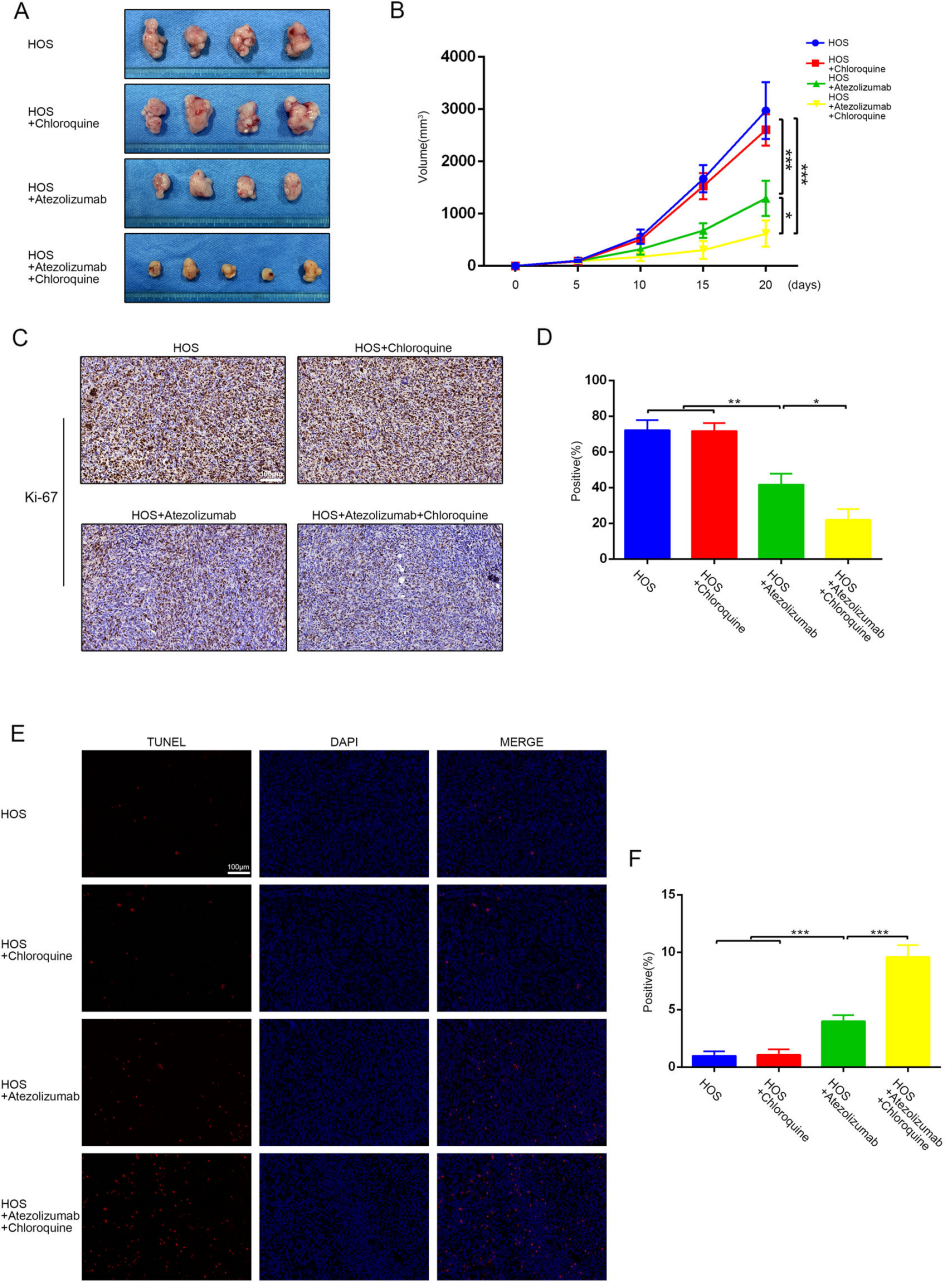
CQ enhances the antitumor effect of atezolizumab on OS in vivo. **A** Images of tumor in each group; **B** Quantification of tumor volume; **C**, **D** Representative IHC staining images and quantification of Ki-67 in each group; **E**, **F** Representative images and quantification of TUNEL staining in each group. All data are from at least three independent experiments and are presented as the means ± SD. *P < 0.05; **P < 0.01; ***P < 0.001.

To examine the antitumor effect of atezolizumab in vivo, we used the xenograft tumor model. Atezolizumab could significantly inhibit the growth of OS when compared with the control group, while no difference in tumor volume was observed between the control group and CQ only group (Fig. 7A, B). Although CQ could not influence the growth of OS alone, the combination of atezolizumab and CQ exerted a more pronounced antitumor effect (Fig. 7A, B), which was consistent with the phenomenon we observed in vitro. Subsequently, we examined the proliferative capacity of OS cells by IHC staining of Ki-67 and found that atezolizumab could suppress the proliferation of OS cells in vivo, and the suppression was further enhanced by the combination of CQ (Fig. 7C, D). Besides, the results of TUNEL staining suggested that atezolizumab promoted apoptosis of OS cells in vivo, and this phenomenon was exacerbated by the addition of CQ (Fig. 7E, F).

**Fig. 8:**
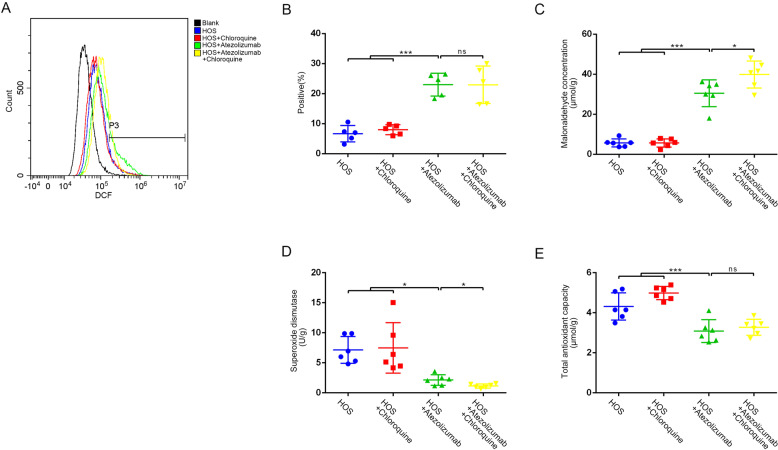
Atezolizumab causes the imbalance between oxidants and antioxidants in vivo. **A**, **B** Release of ROS in vivo was detected by flow cytometry; **C** Level of lipid peroxidation in osteosarcoma was detected by MDA assay; **D** Amount of SOD in osteosarcoma was detected by the total SOD assay kit; **E** Capacity of total antioxidants in osteosarcoma was detected by T-AOC assay kit. All data are from at least three independent experiments and are presented as the means ± SD. *P < 0.05; **P < 0.01; ***P < 0.001.

With further examinations of oxidative stress, we found that atezolizumab could increase the content of MDA while increasing the positive rate of ROS in OS. However, the combination of atezolizumab and CQ only increase the content of MDA in OS but did not further increase the ROS positive rate of OS cells (Fig. 8A–C). The results of antioxidants detection were also consistent with the above results, atezolizumab could decrease T-AOC as well as total SOD in OS (Fig. 8D, E).

Taken together, atezolizumab can break the balance between oxidants and antioxidants and suppress the growth of OS in vivo, and this antitumor effect can be enhanced by the combination of CQ.

## Discussion

Our previous study showed that antibodies against PD-L1 could restore the antitumor immunity of CD8^+^ T cells in OS^12^. In this study, we found that atezolizumab could also induce immune-independent mitochondria-related apoptosis by increasing ROS and cyto-c release in OS. In addition, blocking the protective autophagy induced by atezolizumab could significantly amplify its antitumor effect on OS cells. These findings help us to fully illustrate the antitumor effect of atezolizumab and explore the promising way to further improve its efficacy.

Atezolizumab is a fully-humanized monoclonal antibody against PD-L1, which has achieved satisfactory results in the treatment of advanced tumors^24–26^. Atezolizumab can ameliorate the lymphocyte apoptosis by disrupting the binding of PD-L1 on the surface of tumor to PD-1 on the surface of lymphocytes, and restore the antitumor immunity of lymphocytes, thereby inhibiting tumor growth^27,28^. Whereas, recent studies have shown that the blockade of PD-L1/PD-1 not only had an extracellular effect but also had an important intracellular effect on tumor cells. As previous researches demonstrate that PD-L1 plays a critical role in maintaining stemness and promoting self-renewal as well as tumorigenicity of tumor cells^17,29^, but the specific mechanism is still unclear. Here we discover that atezolizumab can inhibit cell proliferation while inducing apoptosis of OS cells directly in a dose-dependent manner.

To further study the underlying mechanism, we examined the change of intracellular ultrastructure after atezolizumab treatment through transmission electron microscopy. Mitochondria are essential for cells to maintain the physiological state, and it is closely related to the chemotherapy-induced tumor cell apoptosis^30^. We found that the originally uniformly arranged mitochondria crest become dilated, and the whole mitochondria are vacuolated with the addition of atezolizumab, accompanied by impaired mitochondrial function and decreased membrane potential. The damage to mitochondria could increase the permeability of its membrane, which in turn resulted in the excessive release of ROS and cyto-c. Atezolizumab increased the release of ROS while reducing the content of SOD and T-AOC in OS cells, which in turn caused excessive oxidative stress, leading to lipid peroxidation and DNA damage. Meanwhile, the excessive release of cyto-c could bind to APAF-1 in cytoplasm and thus activate caspase-9, one of the initiators of apoptosis, and eventually activating caspase-3 causing mitochondria-related apoptosis in cells^31–33^. The activation of JNK pathway is closely related to the tumor cell apoptosis, especially to the mitochondria-related apoptosis^34,35^. And we propose that the immune-independent cytotoxicity of atezolizumab on OS cells is partially achieved by activating the JNK pathway.

Accumulating evidence indicates mitochondria as the main source for cellular ROS^36^. In the physiological state, there is a certain number of ROS in the body acting as important intracellular messengers to participate in the maintenance of homeostasis^37,38^. But the excessive release of ROS is harmful to the body and therefore activates the protective mechanisms in the body (such as autophagy) to maintain the homeostasis^39^. Autophagy can protect tumor cells from damage induced by external stimuli (protective autophagy), and it can also aggravate the damage caused by external stimuli (destructive autophagy)^40,41^. Given that, a comprehensive understanding of the role of autophagy in tumorigenesis is helpful for better-combined therapy targeting autophagy. In this study, we consider that the activation of autophagy followed by atezolizumab stimulation may be a pro-survival adaptation of tumor cells to resist external stimuli, as the proapoptotic effect of atezolizumab is enhanced after the blockade of autophagy via CQ or siRNA targeting Atg-5 in vitro. In addition, the antitumor effect of atezolizumab combined with CQ is also the most prominent compared with the other groups in the xenograft tumor model. Blocking the conjugation of PD-L1/PD-1 can activate intracellular autophagy^19,42^, and we estimate that the excessive release of ROS induced by atezolizumab may be one of the pathways, by which atezolizumab activates autophagy in OS.

In conclusion, our data indicate that the antitumor effects of PD-L1 antibodies are considerably broader than simply blocking PD-L1/PD-1 conjugation to restore the antitumor immunity of T cells. We unravel a novel mechanism whereby atezolizumab inhibits OS cell proliferation and promotes apoptosis directly in a dose-dependent manner. Additionally, atezolizumab can also activate protective autophagy by damaging mitochondria and causing excessive release of ROS. And blocking protective autophagy can enhance the antitumor effect of atezolizumab. These findings suggest that combining autophagy inhibitor (such as CQ), as a potential adjuvant, with atezolizumab in the treatment of OS may be a promising therapeutic strategy to improve its efficacy.
